# Lessons from a global antimicrobial resistance surveillance network

**DOI:** 10.2471/BLT.22.289520

**Published:** 2023-09-06

**Authors:** Etienne Ruppé, on behalf of the Network for Enhancing Tricycle ESBL Surveillance Efficiency (NETESE) group

**Affiliations:** a Infection, Antimicrobials, Modelling, Evolution (IAME), Institut National de la Santé et de la Recherche Médicale (INSERM), Université Paris Cité and Université Sorbonne Paris Nord, 16 rue Henri Huchard, Paris, F-75870, France.

## Abstract

The World Health Organization developed the Tricycle surveillance programme to obtain a global picture of antimicrobial resistance, especially in countries with limited surveillance capacity. The programme was developed within a One Health perspective. Tricycle provides a framework for applying a standardized technical protocol to determining the prevalence of extended-spectrum β-lactamase (ESBL)-producing *Escherichia coli* in three sectors: the human, animal and environment sectors. Regular use of the protocol would enable information to be obtained on time trends and on inter- and intraregional variations, thereby generating dynamic data on antibacterial resistance for decision-makers. To date, 19 countries have begun implementing the Tricycle protocol, while other countries will start implementation in the coming years. The Network for Enhancing Tricycle ESBL Surveillance Efficiency (NETESE) was established to support countries implementing the Tricycle protocol. Currently, NETESE includes representatives from 15 institutions in eight low- or middle-income countries at different stages of Tricycle protocol implementation, and from four European countries involved in devising the protocol. This paper describes the Tricycle protocol, reports the initial experiences of NETESE participants with its implementation and discusses future challenges and opportunities.

## Résumé

L'Organisation mondiale de la Santé a développé le programme de surveillance Tricycle afin d'avoir une vue globale de la résistance aux antimicrobiens, en particulier dans les pays où les capacités de surveillance sont limitées. Ce programme a été mis au point selon l'approche «One Health». Tricycle établit le cadre d'application d'un protocole technique standardisé qui vise à mesurer la prévalence de souches d'*Escherichia coli* productrices de β-lactamase à spectre étendu (BLSE) dans trois secteurs: humain, animal et environnemental. L'usage régulier du protocole permettrait d'obtenir des informations sur l'évolution dans le temps et les variations inter- et intrarégionales, ce qui générerait des données dynamiques sur la résistance aux antimicrobiens pour les décideurs. À ce jour, 19 pays se sont lancés dans la mise en place du protocole Tricycle, tandis que d'autres prévoient de le faire dans les années à venir. Le réseau NETESE (Network for Enhancing Tricycle ESBL Surveillance Efficiency) a été créé pour les aider à appliquer ce protocole. Actuellement, le réseau NETESE réunit des représentants de 15 institutions dans huit pays à revenu faible ou intermédiaire à différents stades de mise en œuvre du protocole Tricycle, ainsi que quatre pays européens impliqués dans sa conception. Le présent document décrit le protocole Tricycle, partage les premières expériences des participants au réseau NETESE en matière de déploiement, et aborde les futurs défis et opportunités qui y sont liés.

## Resumen

La Organización Mundial de la Salud desarrolló el programa de vigilancia Tricycle para obtener un panorama global de la resistencia a los antimicrobianos, especialmente en países con una capacidad de vigilancia limitada. El programa se creó desde la perspectiva One Health. Tricycle proporciona un marco que permite aplicar un protocolo técnico estandarizado con el fin de determinar la prevalencia de *Escherichia coli* productora de β-lactamasa de espectro extendido (ESBL) en tres sectores: el humano, el animal y el medio ambiente. El uso habitual del protocolo permitiría obtener información sobre las tendencias temporales y las variaciones inter e intrarregionales, generando así datos dinámicos sobre la resistencia a los antibacterianos que resultarían útiles para los responsables de la toma de decisiones. Hasta la fecha, 19 países han comenzado a implementar el protocolo Tricycle, mientras que otros países comenzarán a aplicarlo en los próximos años. La red NETESE (Network for Enhancing Tricycle ESBL Surveillance Efficiency), se creó para apoyar a los países que implementan el protocolo Tricycle. Actualmente, NETESE está formada por representantes de 15 instituciones de ocho países con ingresos medios o bajos, que se encuentran en diferentes etapas de implementación del protocolo Tricycle. También incluye a representantes de cuatro países europeos que intervienen en el diseño del protocolo. El presente documento describe el protocolo Tricycle, recoge las primeras experiencias de los participantes de NETESE durante la implementación de dicho protocolo, y aborda tanto los retos como las oportunidades futuras.

## ملخص

قامت منظمة الصحة العالمية بتطوير برنامج الرصد Tricycle لتشكيل صورة عالمية لمقاومة مضادات الميكروبات، وبخاصة في الدول ذات القدرة المحدودة على الرصد. تم تطوير البرنامج من خلال منظور Tricycle. يوفر برنامج One Health إطار عمل لتطبيق بروتوكول تقني موحد لتحديد مدى انتشار الإشريكية القولونية المعوية المنتجة لبكتيريا بيتا لاكتيماز ذات النطاق الممتد الموسع ( ESBL) في ثلاثة قطاعات: قطاعات الإنسان، والحيوان، والبيئة. إن الاستخدام المنتظم للبروتوكول سوف يتيح الحصول على معلومات عن الاتجاهات الزمنية، وعن التباينات بين الأقاليم وداخلها، وبالتالي توليد بيانات ديناميكية عن مقاومة مضادات البكتيريا لصانعي القرار. وحتى الآن، بدأت 19 دولة في تنفيذ بروتوكول Tricycle، بينما بدأت دول أخرى في التنفيذ في السنوات المقبلة. إن شبكة تعزيز كفاءة رصد برنامج Tricycle لبكتيريا بيتا لاكتيماز ذات النطاق الممتد الموسع ( NETESE)، تم إنشاؤها لدعم الدول التي تقوم بتنفيذ بروتوكول Tricycle. تضم شبكة NETESE حاليًا ممثلين من 15 مؤسسة في ثمانية دول ذات دخل منخفض أو دخل متوسط، في مراحل مختلفة من تنفيذ بروتوكول Tricycle، ومن أربع دول أوروبية مشاركة في وضع البروتوكول. تصف هذه الورقة بروتوكول Tricycle، وتشير إلى التجارب الأولية للمشاركين في شبكة NETESE خلال تنفيذها، وتناقش التحديات والفرص المستقبلية.

## 摘要

世界卫生组织制定了 Tricycle 监测计划，以了解全球抗菌药物的耐药性情况，特别是在监测能力有限的国家。该计划根据“同一健康”的理念制定。，Tricycle 为应用标准化技术方案在三个领域【人类、动物和环境方面】中确定产超广谱β-内酰胺酶 (ESBL) *大肠杆菌*的流行率提供了一个框架。定期实施该方案将能够及时获得其相关趋势以及区域间和区域内变化的信息，从而为决策者生成抗菌药物耐药性的动态数据。迄今为止，19个国家已开始实施 Tricycle 方案，而其他国家将在未来几年开始实施。建立了提高 Tricycle ESBL 监测效率的网络 (NETESE)，以支持实施 Tricycle 方案的国家。目前，NETESE 包括处于 Tricycle 方案实施不同阶段的八个低收入或中等收入国家的 15 个机构的代表以及参与该方案制定的四个欧洲国家代表。本论文介绍了 Tricycle 方案，报告了 NETESE 参与者实施该方案的初步经验，并讨论了未来会面临的挑战和机遇。

## Резюме

Всемирной организацией здравоохранения была разработана программа эпиднадзора Tricycle для получения глобальной картины устойчивости к противомикробным препаратам, особенно в странах с ограниченными возможностями эпиднадзора. Программа разрабатывалась в рамках концепции «Единое здоровье». Tricycle представляет собой основу для применения стандартизированного технического протокола для определения распространенности штамма *Escherichia coli*, продуцирующей β-лактамазы расширенного спектра (extended-spectrum β-lactamase, ESBL), в трех секторах: человеческом, животном и окружающей среде. Регулярное использование протокола позволит получать информацию о временных тенденциях, межрегиональных и внутрирегиональных изменениях и таким образом формировать динамические данные об антибактериальной устойчивости для лиц, принимающих решения. На сегодняшний день 19 стран приступили к внедрению протокола Tricycle. Другие страны начнут внедрение в ближайшие годы. Для поддержки стран, внедряющих протокол Tricycle, была создана сеть по повышению эффективности надзора за бета-лактамазами расширенного спектра действия Tricycle (Network for Enhancing Tricycle ESBL Surveillance Efficiency, NETESE). В настоящее время в состав NETESE входят представители 15 учреждений из восьми стран с низким и средним уровнем доходов, находящихся на разных стадиях внедрения протокола Tricycle, а также представители четырех европейских стран, участвовавших в разработке протокола. В данной работе приводится описание протокола Tricycle, сообщается о первом опыте его применения участниками NETESE и обсуждаются дальнейшие задачи и возможности.

## Introduction

To provide a picture of antimicrobial resistance in humans, animals and the environment in all countries, especially those with limited surveillance capacity, the World Health Organization (WHO) Advisory Group on Integrated Surveillance of Antimicrobial Resistance developed the Tricycle protocol for global antimicrobial resistance surveillance.^1^ Tricycle provides a framework for applying a standardized technical protocol to determine the prevalence of *Escherichia coli* that produce extended-spectrum β-lactamases in three sectors: the human, animal and environment sectors. To do this, extended-spectrum β-lactamase-producing *E. coli* are sought in: (i) the faeces of pregnant women; (ii) the blood of people with bloodstream infections; (iii) chicken caeca from wet markets or slaughterhouses; and (iv) water specimens from rivers upstream and downstream, wet markets and human wastewater.

The Advisory Group on Integrated Surveillance of Antimicrobial Resistance decided to monitor extended-spectrum β-lactamase-producing *E. coli* because: (i) it is present in the three sectors; (ii) its prevalence in humans varies widely both within and between countries; and (iii) it is responsible for a substantial burden of infection.^2^ Hence, extended-spectrum β-lactamase-producing *E. coli* was deemed to be a relevant and representative proxy for the magnitude of, and trends in, antimicrobial resistance; though, it may not accurately reflect the overall global problem of antimicrobial resistance. Nevertheless, as this approach includes antimicrobial resistance surveillance in animals and the environment, its findings can be linked to WHO’s Global Antimicrobial Resistance and Use Surveillance System, which reports *E. coli* resistance to third-generation cephalosporins, among many other antimicrobial-pathogen combinations.^3^ Moreover, regular use of a standard protocol enables information to be obtained on time trends and on inter- and intraregional variations, thereby generating dynamic data on antibacterial resistance for decision-makers. WHO’s role is: (i) to continue supporting countries in implementing the Tricycle protocol; (ii) to evaluate the implementation of the protocol and expand its use; and (iii) to support national data collection from human, animal and environment sectors and the incorporation of new data into a global repository.

To date, 19 countries have begun implementing the Tricycle protocol under the sponsorship of the Fleming Fund in the United Kingdom of Great Britain and Northern Ireland, and the Fondation Mérieux in France. Additional countries will be included in the coming years. However, no formal procedure has been developed to support countries wishing to implement the protocol. We, therefore, took advantage of an open call for transnational networks from the Joint Programming Initiative on Antimicrobial Resistance in 2018 to fill this gap, and created the Network for Enhancing Tricycle ESBL [extended-spectrum β-lactamase] Surveillance Efficiency (NETESE).^4^ Currently, NETESE includes representatives from eight low- or middle-income countries at different stages of Tricycle protocol implementation, and from four European countries instrumental in its development and implementation. The network was established through two meetings: one in Paris, France, in 2019 and one in Brussels, Belgium, in 2022. Between these meetings, participants interacted through quarterly virtual conferences on specific topics of interest and via a social network group. 

Here we describe the initial achievements of NETESE, discuss remaining challenges to the implementation of the Tricycle protocol and anticipate its future. We do not describe the results of previous campaigns using the Tricycle protocol in detail. Instead, we report the experience of the network, which we believe can be valuable for organizations wishing to implement the protocol. Of note, we do not describe difference in performance between network members but rather the potential difficulties encountered in applying the protocol.

## The Tricycle protocol

Full details of the Tricycle protocol are available from the WHO website.^1^ Briefly, the protocol is a surveillance programme aligned with the One Health approach that considers human, animal and environment sectors within the same time window and geographical area.^5^ There are six core work packages: package 1: human (hospital and community); package 2: food chain; package 3: environment; package 4: molecular characterization; package 5: epidemiology design and analysis; and package 6: management and coordination. 

In addition, there are two optional work packages: package 7: linkage with WHO’s Global Antimicrobial Resistance and Use Surveillance System; and package 8: links with antimicrobial consumption and use surveillance systems. For an initial surveillance campaign, the protocol recommends starting in the largest, or capital, city of a country and expanding over time, depending on the resources available.

Currently, 19 countries (not all in NETESE) are at various stages of implementing the Tricycle protocol. Six hosted pilot studies (Ghana, Indonesia, Madagascar, Malaysia, Pakistan and Senegal); six have already implemented the protocol (Burkina Faso, Cameroon, India, Jordan, Nepal and Zimbabwe); and seven are planning to implement it (Côte d'Ivoire, Democratic Republic of the Congo, Islamic Republic of Iran, Morocco, Nigeria, Sudan and Zambia). Noteworthy is that eight of the 19 countries are part of NETESE (Table 1). This article pertains to the experience of these eight countries. All 19 countries have written a national action plan on antimicrobial resistance, as have 149 other countries. Table 1 lists the countries in NETESE and their contact persons. 

**Table 1:** NETESE participants and their Tricycle protocol implementation stage, 2023

Country	Organization or affiliation	Individual NETESE member	Tricycle protocol implementation stage
Burkina Faso	Centre Hospitalier Universitaire Sourô Sanou, Bobo-Dioulasso	Abdoul-Salam Ouedraogo	Campaign to be scheduled
Cameroon	Department of Laboratory Medicine, Microbiology, Parasitology, Haematology and Infectious Diseases, Faculty of Medicine and Biomedical Sciences, University of Yaoundé, Yaoundé	Francois Xavier Mbopi Keou	One campaign (2022)
Côte d'Ivoire	Institut Pasteur de Côte d’Ivoire, Abidjan	Valérie Gbonon M Bengue	Campaign scheduled for 2023
Democratic Republic of the Congo	Institut National de Recherche Biomédicale, Ministry of Public Health, Kinshasa	Berthe-Noëlle Miwanda and Emmanuel Busha	One campaign (2021)
Indonesia	Centre for Research and Development of Biomedical and Basic Health Technology, National Institute of Health Research and Development, Jakarta	Nelly Puspandari	One pilot study (2018–2019)
Madagascar	Centre d’Infectiologie Charles Mérieux, Faculty of Medicine, University of Antananarivo, Antananarivo	Luc Samison and Milen Milenkov	Three campaigns (2019, 2020, 2022)
Malaysia	Medical Care Quality Section, Medical Development Division, Ministry of Health, Kuala Lumpur	Suraya bt Amir Husin	Two campaigns (2018, 2022)
Senegal	Institut Pasteur Dakar, Dakar	Bissoume Sambe	One pilot study (2018–2019) and one campaign (2022)

NETESE: Network for Enhancing Tricycle ESBL [extended-spectrum β-lactamase] Surveillance Efficiency.

In work package 1 (the human sector), the Tricycle protocol focuses on two measures: (i) the proportion of *E. coli* bloodstream infections involving extended-spectrum β-lactamase-producing *E. coli* (expected sample size: 100); and (ii) the proportion of healthy pregnant women carrying extended-spectrum β-lactamase-producing *E. coli* in faeces (expected sample size: 100). Two main issues related to these measures were encountered by NETESE members. First, the proportion of blood cultures that were positive varied greatly across countries, which led to the identification of two patterns (Table 2). The first pattern was characterized by a low blood culture positivity rate, with a high proportion of blood cultures positive for *E. coli* (e.g. in Malaysia). This pattern is similar to that observed in tertiary care hospitals in Europe, where blood culture is frequently performed, even for patients with community-acquired infections. The second pattern was characterized by a high blood culture positivity rate, with a low proportion of blood cultures positive for *E. coli* (e.g. in Indonesia, Madagascar and Senegal), which possibly indicates that blood culture was reserved mainly for hospital-acquired infections. This observation raised concerns about comparing the frequency of extended-spectrum β-lactamase-producing *E. coli* in blood cultures across different countries, because there could be substantial differences in the populations sampled. Additionally, the cost of blood culture was not covered by the budget provided by funders, which could have limited its use in low-resource settings. Consequently, the possibility of using alternative specimens, such as urine, was considered.

**Table 2:** Blood culture results, Tricycle antimicrobial resistance surveillance programmes, Indonesia, Madagascar, Malaysia and Senegal, 2018–2022

Country	No. blood samples	No. blood samples with positive blood cultures (%)	No. blood samples with blood cultures positive for *E. coli* (%)	Proportion of samples with positive blood cultures that were positive for *E. coli* (%)^a^
Indonesia	6 883	1342 (19.5)	100 (1.5)	7.5
Madagascar	1 150	310 (27.0)	20 (1.7)	6.5
Malaysia	38 300	1183 (3.1)	318 (0.8)	26.9
Senegal	219	63 (28.8)	5 (2.2)	7.9

*E. coli: Escherichia coli*.

^a^ Each figure in this column was derived from the number in the fourth column divided by the number in the third column.

The second issue was the challenge of obtaining faecal specimens from pregnant women, and we agreed that rectal swabs could serve as acceptable substitutes, although there were also difficulties in obtaining rectal swabs. To encourage participation, it was preferable to provide information about the Tricycle protocol individually to pregnant women during prenatal consultations or at the time of delivery, rather than to provide information to groups of women, typically in waiting rooms. In both cases, the commitment of hospital staff was crucial. Informed consent was obtained without difficulty from all pregnant women in countries participating in NETESE, as required by the Tricycle protocol (sections 4.1.2.3 and 4.1.2.5).^1^

For work package 2 (the food animal sector), chicken was chosen as the host species for several reasons. First, people around the world widely consume chicken meat, there are few religious restrictions. Second, chicken production encompasses various conditions, ranging from highly intensive farming through medium-intensive and pastoral farming, to backyard and household settings. Third, chicken is readily accessible for sampling. Lastly, some countries have already implemented surveillance systems that involve collecting chicken caecal samples. The recommended sampling site is a wet market, where chickens should be purchased from multiple stalls representing different flocks (expected sample size: 240). After purchase, it is recommended that chickens are separated from each other to prevent cross-contamination during transportation, as advised by NETESE participants.

In work package 3 (the environment sector), hotspot sources such as sewage, and wastewater from slaughterhouses and wet markets should be monitored for extended-spectrum β-lactamase-producing *E. coli*. In addition, rivers that receive wastewater from these sources should be sampled both upstream and downstream. Noteworthy is that extended-spectrum β-lactamase-producing *E. coli* are not only detected in this process but also quantified. Furthermore, samples should be obtained from another city in the country with approximately 100 000 inhabitants in addition to the largest, or capital, city to ensure samples are representative of cities of a similar size in that country. No specific difficulties related to work package 3 were raised during NETESE meetings.

Overall, NETESE participants found the Tricycle laboratory protocol straightforward and easy to follow. Participants also recognized that the training received in some countries (e.g. in the Kingdom of the Netherlands and Indonesia in 2017, and in Jordan in 2019) was highly beneficial. However, limitations on the availability of laboratory consumables posed a major challenge for implementation. The high cost of consumables and the difficulties encountered during importation were partly responsible. Furthermore, even when consumables were finally delivered, their expiration dates could be relatively soon. To facilitate implementation, WHO provided laboratory consumables to most countries applying the Tricycle protocol. Additionally, WHO is working towards including laboratory consumables in its internal procurement catalogue for WHO staff, with the aim of making those consumables more accessible.

The estimated minimum budget required for implementing the Tricycle protocol was approximately 25 000 euros (€) (29 000 United States dollars, US$) per campaign, which includes sampling human, animal and environment sectors. However, the estimated expenditure in existing campaigns was higher, at around €50 000 (US$ 58 000), with the additional amount varying according to staff costs in each sector. Noteworthy is that the success of each campaign relied on the commitment of staff at different levels in the three sectors. In some cases, financial incentives were necessary to cover the additional workload and to ensure staff were committed to the programme. There were various sources of funding. In Indonesia, WHO provided funding for a 6-month pilot study and a second surveillance campaign.^6^ In Madagascar, the first two campaigns were supported by the Fondation Mérieux,^7^^,^^8^ whereas the third campaign will be funded by the Joint Programming Initiative on Antimicrobial Resistance. Local people involved in the campaigns felt that additional support will be required through national action plans on antimicrobial resistance to ensure Tricycle protocol campaigns are sustainable.

## Early results

**Fig. 1:**
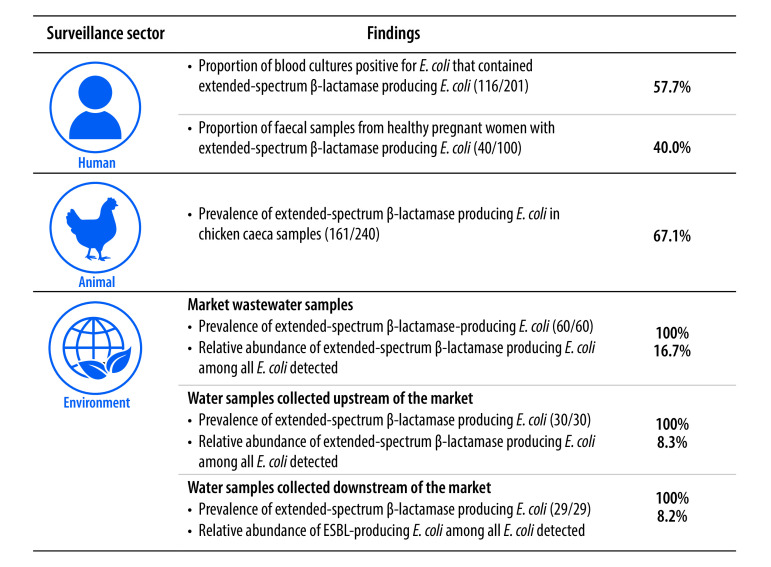
Main results of a Tricycle protocol pilot study, Indonesia, 2018–2019^6^

To date, publications from Ghana, Indonesia and Madagascar have reported the results of implementing the Tricycle protocol,^6^^–^^9^ either in one surveillance sector or in all three sectors. In Ghana, the prevalence of extended-spectrum β-lactamase-producing *E. coli* was remarkably high in the environment sector, specifically in two rivers that received human and animal effluent: 98% (94/96) of samples tested positive for extended-spectrum β-lactamase-producing *E. coli*.^9^ In Madagascar, the overall prevalence of extended-spectrum β-lactamase-producing *E. coli* in rectal swabs from 492 pregnant women across four different regions of the country was 34% (range between regions: 12% to 65%).^7^ Interestingly, a risk factor analysis revealed that the risk of extended-spectrum β-lactamase-producing *E. coli* carriage was higher in the rainy season (odds ratio: 2.9; 95% confidence interval: 1.3–5.6). In addition, whole-genome sequencing indicated that human-to-human transmission had occurred. The findings for all three sectors of a pilot Tricycle protocol campaign in Indonesia are summarized in Fig. 1:^6^ (i) the prevalence of extended-spectrum β-lactamase-producing *E. coli* in faecal samples from pregnant women was 40.0% (40/100); (ii) the prevalence in samples from broiler chickens was 67.1% (161/240); and (iii) the prevalence in all water specimens, which were collected upstream and downstream from cities and from markets, was 100% (119/119).

*E. coli: Escherichia coli*.

## Future prospects

### Mini-lab

To overcome problems with the availability of laboratory consumables, in 2019 *Médecins Sans Frontières* launched a mini-lab: a transportable, self-contained, quality-assured, stand-alone, clinical bacteriology laboratory that can be operated by inexperienced technicians and used in low-resource settings.^10^ The mini-lab comprises six numbered and colour-coded bench-boxes. Each box unfolds into a fully equipped and ready-to-use workbench and contains equipment (e.g. a microscope or incubator), consumables, documentation and informational material. With the mini-lab, bloodstream infections can be diagnosed by identifying pathogens using biochemical tests, and their antibiotic susceptibility can be assessed using the microbroth dilution method. Consumables are robust in warm and humid conditions and have a long preservation time. The Democratic Republic of the Congo is currently assessing the use of the mini-lab to diagnose bloodstream infections.

### Communication of results

Implementation of the Tricycle protocol requires the involvement of WHO at national, regional and global levels. National authorities can request support for implementing the Tricycle protocol through the WHO country office, which will subsequently coordinate with regional and global bodies. Teams involved in Tricycle protocol surveillance campaigns are encouraged to publish their results and to present them at national and international conferences.^6^^,^^7^^,^^9^ Results should also be presented to national agencies from the human, animal and environment sectors, with the aim of increasing government support.

In the community, pregnant women were initially proposed as ambassadors of antimicrobial resistance because they are individually informed about this public health problem when they are included in surveillance programmes. However, as currently there is no means of bacterial decolonization for patients with intestinal extended-spectrum β-lactamase-producing *E. coli*, any communication about antimicrobial resistance should be accompanied by concrete actions, such as better use of antimicrobials or sanitary measures. Moreover, informing patients that they are carrying extended-spectrum β-lactamase-producing *E. coli* could result in unnecessary anxiety. Hence, it was decided that pregnant women should not be informed about carrying extended-spectrum β-lactamase-producing *E. coli*, though clinicians should be informed so that antimicrobial regimens can be adapted when there is an infection.

### Extending the Tricycle protocol

The Tricycle protocol can be adapted to include additional satellite surveillance or research project protocols on antimicrobial resistance. As the prevalence of carbapenemase-producing Enterobacterales is rising in the community in many regions,^11^ these bacteria could complement extended-spectrum β-lactamase-producing *E. coli* as another emerging indicator of antimicrobial resistance in the Tricycle protocol. This approach is the rationale for a project funded by the Joint Programming Initiative on Antimicrobial Resistance – the TRIuMPH project, which stands for Tricycle protocol: upscaling to national monitoring and detection of carbapenemase-producing Enterobacterales and whole-genome sequencing pipelines for One Health surveillance. The TRIuMPH project adds new protocols and research elements to the Tricycle protocol, mainly involving the detection of carbapenemase-producing Enterobacterales in addition to extended-spectrum β-lactamase-producing *E. coli* and the application of whole-genome sequencing. This project includes NETESE partners Madagascar and Malaysia, Pakistan, and organizations that contributed to Tricycle protocol development, namely the *Institut national de la santé et de la recherche médicale* (INSERM) in France; and Utrecht University and the National Institute for Public Health and the Environment (RIVM) in the Kingdom of the Netherlands. Also, the TRIuMPH project aims to include surveillance of more than one city in each country, as currently recommended by the Tricycle protocol. In fact, the Tricycle protocol promotes a stepwise approach that enables countries to progressively incorporate additional cities, provinces, sectors, subsectors and bacterial pathogens.

### Other bacteria and specimens

Currently, the Tricycle protocol includes additional elements that are optional and remain outside the core protocol: (i) antimicrobial usage assessment; (ii) epidemiological studies; (iii) antimicrobial residue assessment; (iv) drinking water analysis; and (v) hospital wastewater analysis. For water specimens, extended-spectrum β-lactamase-producing *E. coli* is still the indicator of choice. During NETESE meetings, participants suggested that specimens could be obtained from other sources: for example, popular foods such as oranges and eggs. In addition, it was also discussed whether other bacterial species, such as *Salmonella* spp., *Campylobacter* spp. or *Helicobacter pylori*, could be monitored. The conclusion was that the core Tricycle protocol should be applied, and that other options could be considered depending on local interest, funding and technical capabilities.

### Genome sequencing

The Tricycle protocol recommends that the genomes of extended-spectrum β-lactamase-producing *E. coli* should be sequenced, when feasible, to gain a better understanding of the interconnectedness between human, animal and environment sectors and antimicrobial resistance genes.^12^ Previous cross-sector studies of the genetic relatedness of multidrug-resistant *E. coli* in high-income settings revealed that animal and human strains were largely distinct.^13^^,^^14^ This finding was attributed to stringent hygiene standards that limited the circulation of multidrug-resistant *E. coli* between animals and humans. However, in low-resource settings, where living conditions are not conducive to the same level of hygiene, intersectoral circulation of multidrug-resistant *E. coli* could be more common. In a recent study of the Tricycle surveillance campaign in Madagascar, researchers observed both the circulation of *E. coli* clones and the transmission of plasmids carrying antibiotic resistance genes between sectors.^8^ This finding highlights the importance of using long-read sequencing for genomic analysis in the One Health context. Although access to genome sequencers and bioinformatic facilities may pose challenges in certain settings, initiatives such as SeqAfrica,^15^ which is supported by a regional grant from the Fleming Fund, offer opportunities for NETESE participants to collect *E. coli* strains and perform genomic sequencing.

### Sustainability

The sustainability of Tricycle campaigns depends on funding. To date, only two countries (Madagascar and Malaysia) have conducted more than one campaign. Clearly, annual longitudinal sampling using the same protocol would provide more valuable information than single campaigns. However, although different funding sources have been used for first-time campaigns, these will not be available over the longer term. Hence, alternative, sustained funding sources must be secured to ensure Tricycle campaigns continue into the future. With this end in view, Tricycle campaign participants were encouraged to ask governments for long-term support. Communicating the results of Tricycle campaigns to stakeholders will be pivotal in achieving this. Moreover, a Tricycle campaign can serve as the first step in establishing a national programme on integrated surveillance because it: (i) creates a holistic collaboration between human, animal and environment sectors and subsectors; (ii) ensures laboratories have the capacity to culture, isolate, identify and characterize the relevant pathogens; and (iii) mobilizes human and financial resources.

## Conclusion

The aim of the Tricycle protocol was to provide a simple, integrated approach to antimicrobial resistance surveillance across human, animal and environment sectors. The protocol adopts a stepwise approach with the end goal of establishing comprehensive national surveillance systems. Importantly, the Tricycle protocol provides data that can be used to make national, regional and global comparisons. Moreover, the data can be combined with information on antimicrobial use and consumption in both human and animal sectors, which countries collect in accordance with guidance issued by WHO and the World Organisation for Animal Health.^3^ Access to this integrated information will increase our understanding of the extent of antimicrobial resistance globally, and facilitate the development of containment strategies. 

Crucially, implementation of the Tricycle protocol fosters multisectoral collaboration on antimicrobial resistance surveillance, as promoted and supported by the Quadripartite Collaboration on the One Health approach, which comprises the Food and Agriculture Organization, the United Nations Environment Programme, the World Organisation for Animal Health and WHO.^16^ We hope this collaboration can be helpful in advocating for increased government commitment, support and capacity-building on antimicrobial resistance surveillance.
